# Endobronchial Suture of Tracheoesophageal Fistula Through Rigid Bronchoscopy Without Tracheostomy: A Preliminary, Observational Retrospective Study

**DOI:** 10.3390/jcm14010110

**Published:** 2024-12-28

**Authors:** Giovanni Galluccio, Vito D’Agnano, Ilaria Menichini, Antonio Giulio Napolitano, Umberto Masi, Andrea Bianco

**Affiliations:** 1 Centre for Thoracic Endoscopy and Interventional Pulmonology, Regina Apostolorum Hospital, 00041 Rome, Italy; ilariamenichini@gmail.com (I.M.); antoniogiulionapolitano@gmail.com (A.G.N.); 2 Unit of Thoracic Surgery, Azienda Ospedaliera San Camillo-Forlanini, Carlo Forlanini Hospital, 00151 Rome, Italy; 3 Department of Translational Medical Sciences, University of Campania L. Vanvitelli, 80131 Naples, Italy; vito.dagnano@studenti.unicampania.it (V.D.); umberto.masi@studenti.unicampania.it (U.M.); andrea.bianco@unicampania.it (A.B.); 4 U.O.C. Clinica Pneumologica L. Vanvitelli, Monaldi Hospital, A.O. dei Colli, 80131 Naples, Italy; 5 Division of Pneumology, A. Cardarelli Hospital, 80131 Naples, Italy

**Keywords:** tracheoesophageal fistula, bronchoscopy, rigid bronchoscopy, endoscopic surgery

## Abstract

**Background:** A tracheoesophageal fistula (TEF) represents a condition characterized by abnormal communication between the gastrointestinal tract and the airways. Although the current gold-standard treatment is surgery, pre-existing clinical conditions may represent contraindications. We therefore propose a bronchoscopic approach through rigid bronchoscopy without tracheostomy for total repair in patients suffering from benign tracheoesophageal fistulas. **Methods**: Fistula suture through rigid bronchoscopy with either absorbable (Vycryl 3.0, Ethicon, Inc.) or non-absorbable (Prolene, Ethicon US, LLC. 2022.) sutures was performed using a long needle holder as an alternative resolutive procedure to surgery. From 2015 to 2022, we retrospectively reviewed 10 consecutive patients affected by TEFs in our Endoscopic Unit at San Camillo-Forlanini Hospital (Rome, Italy) who underwent this bronchoscopic procedure. The coprimary outcomes were the proportion of fistulas successfully treated with the innovative treatment proposed and the rate of procedure-related complications. **Results**: The complete healing of the fistula was achieved in nine of the ten patients after 1 year. Follow-up with flexible bronchoscopy was scheduled and carried out at 1, 3, and 12 months following rigid bronchoscopy. Overall, seventeen endoscopic repair procedures were performed. Five of these patients required more than one endoscopic treatment to reach complete fistula closure. Fistula closure was not achieved post-procedure in one patient. No complications during procedures or in the follow-up period were reported. **Conclusions**: Despite the small cohort, our preliminary study has demonstrated that the endoscopic approach through rigid bronchoscopy, without tracheostomy, represents a safe and satisfactory alternative for patients affected by TEFs who are not suitable for surgery.

## 1. Introduction

A tracheoesophageal fistula (TEF) represents an abnormal, fistulous connection between the proximal and/or distal esophagus and the airway. TEFs are generally classified into two major categories: congenital and acquired. TEFs—either acquired or congenital—are clinically characterized by the continuous spillover of oral and gastric secretions into the airways and pose great challenges for clinicians in terms of both diagnosis and treatment [1].

**Figure 1:**
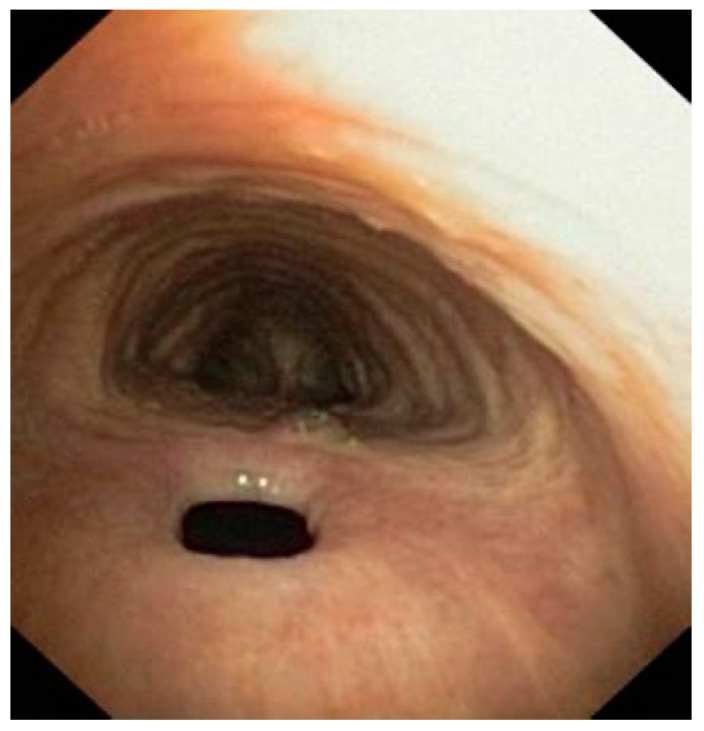
Endoscopic image of an acquired, post-intubation tracheoesophageal fistula in a 46-year-old male.

In adults, an acquired fistula (Figure 1) can be either benign or malignant. Benign causes include post-traumatic and postsurgical causes and those related to inflammatory diseases. The majority of malignant TEFs are caused by either esophageal or lung cancer. In addition to the neoplastic infiltration of the wall, chemo- or radiotherapy treatment may contribute to TEF development.

Patients generally present with relapsing or recurrent pneumonia due to the aspiration of gastric fluid and increased tracheal secretions. A pathognomonic sign of a TEF is sudden gastric distension with low tidal volume values in a patient under positive-pressure mechanical ventilation, while extubated patients may complain of a choking sensation during swallowing (Ono’s sign); this symptom is due to the patient’s position and may be exacerbated in the left lateral decubitus or standing position. As fistulas develop from the anterior wall of the esophagus in most cases, patients assume a supine position to reduce coughing (assuming a supine position reduces coughing). Diagnosis involves an X-ray with a barium swallow test followed by computed tomography, and confirmation requires endoscopy. The closure of both tracheal and esophagus leaks by surgical repair is recommended when feasible. However, in malignant TEFs, the compromised clinical status and coexistence of comorbidities may represent major contraindications in the surgical approach [2,3].

Advances in endoscopic treatment have recently emerged as a potential approach in TEFs. The most widely used treatment involves the placement of an esophageal or tracheal stent; covered self-expanding metal stents (SEMSs) have been emerging as a superior option to non-expandable esophageal stents. Covering materials—silicone, polyurethane, and polytetrafluoroethylene—have been shown to reduce the re-intervention rate compared to uncovered stents [2]. A combined esophageal and tracheal stent may be considered when the fistula trait is larger than 2 cm, as esophageal stenting may compromise airway patency, and in the case of pre-existing tracheal stenosis [2,4]. The clinical success rate of stents is reported to range from 60 to 80% depending on the size, anatomy, and type of the TEF [5,6,7]. However, in some cases—such as fistulas in the middle or lower portion of the esophagus—stent placement is contraindicated. Furthermore, the insertion of a tracheal stent represents a palliative approach and commits the patient to frequent bronchoscopies for the removal of secretions, which may pose difficulties in particularly compromised patients.

In 2001, Ambrogi and coworkers proposed, with good results, a double suture of the esophagus and trachea using a longitudinal incision in the anterior wall of the trachea [8]. Following their study, we treated a number of patients in our endoscopy department using a modified Angeletti’s technique involving the use of a rigid bronchoscope to aspirate secretions and insert an optic for vision, while the suture of the TEF was carried out through a tracheostomy [9].

The objective of the present retrospective, observational study is to present an innovative technique that consists of the total endoscopic repair of a non-neoplastic TEF, without the need for a tracheostomy. The coprimary outcomes were the proportion of fistulas successfully treated with the innovative treatment proposed and the rate of procedure-related complications.

## 2. Materials and Methods

### 2.1. Patients

A retrospective monocentric study was conducted in a cohort population admitted to the Endoscopic Unit at San Camillo-Forlanini Hospital (Rome, Italy) between January 2015 and December 2022. Pre-operative demographic and clinical data were collected. Informed consent was obtained from each patient prior to their undergoing each procedure, which was conducted according to Local Ethical Committee guidance. The study was conducted in accordance with the Declaration of Helsinki. This retrospective study has been reported in accordance with the Strengthening The Reporting Of Cohort, Cross-Sectional and Case–Control Studies in Surgery Guidelines 2021 (STROCSS) [10]. The study was retrospectively registered with the Research Registry (https://www.researchregistry.com), with the following identifying number: researchregistry10720 (24 September 2024).

### 2.2. Intervention and Follow-Up

#### 2.2.1. Pre-Operative Assessment

All patients underwent a pre-operative anesthetic assessment, and an anesthetist attended all procedures. Procedures were conducted under deep sedation with propofol (induction dose: 2–2.5 mg/kg) but with spontaneous ventilation. All patients underwent pre-operative flexible bronchoscopy in order to define the fistula characteristics.

Fistulas characterized by mucosal hyperemia, congestion, fringed margins, and fragile mucosa with easy bleeding at inspection were classified as early. Conversely, fistulas were classified as non-early if they were characterized by well-demarcated margins, pale mucosa, and scarring.

#### 2.2.2. Procedure Description

**Figure 2:**
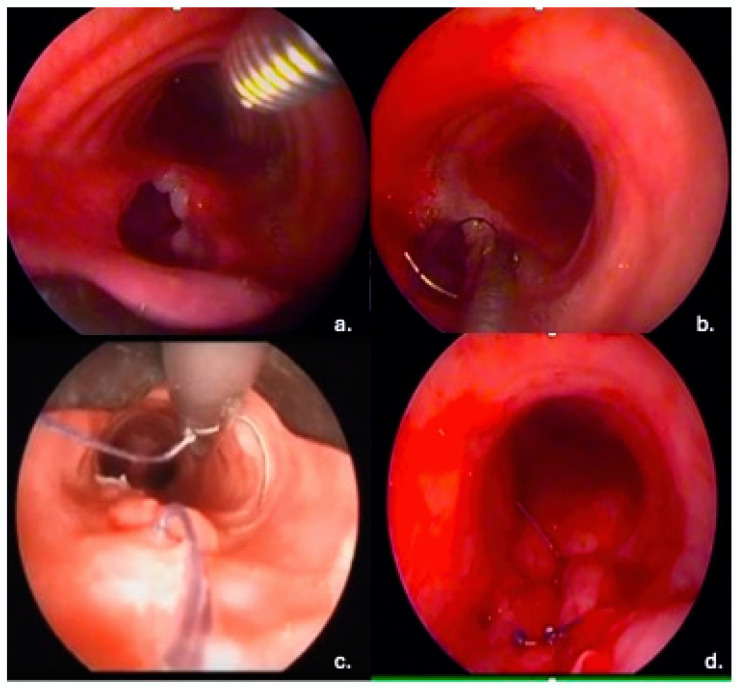
(**a**–**d**): Suture of a tracheoesophageal fistula during rigid bronchoscopy. (**a**) The fistula is exposed through rigid bronchoscopy. (**b**) The needle is angled through the needle holder, and the needle tip is passed through the margin of the fistula. (**c**) The needle tip and the wire are picked up through the needle holder. (**d**) The fistula is sutured, and the surgical threads are cut.

All patients underwent a rigid bronchoscopy. The operating team was composed of a pulmonologist, who led the procedures, supported by another pulmonologist, an anesthesiologist, one scrub nurse, and one operating room nurse. A large tracheal tube (Harrell Dumon © Tracheoscope, 13.4 mm in diameter, Pembroke, MA, USA) was inserted into the trachea and connected to the oxygen. A rigid aspirator and a rigid telescope (Storz) were inserted into the tracheoscope (Figure 2a). A 3-0 curved needle was inserted into the tracheoscope through a needle holder. At the level of the fistula, the needle was angled and the needle tip passed through the margins of the fistula (Figure 2b). The needle, along with the wire, was subsequently picked up by means of the needle holder (Figure 2c). A slipknot (generally a Roeder knot) was made outside of the tracheoscope and pushed down with a long Knot Pusher (Storz) to bring the fistula edges together. The number of knots needed ranged from 1 to 3, according to the shape and size of the fistula. The surgical thread was cut by means of a contact Nd–YAG laser (Figure 2d).

**Figure 3:**
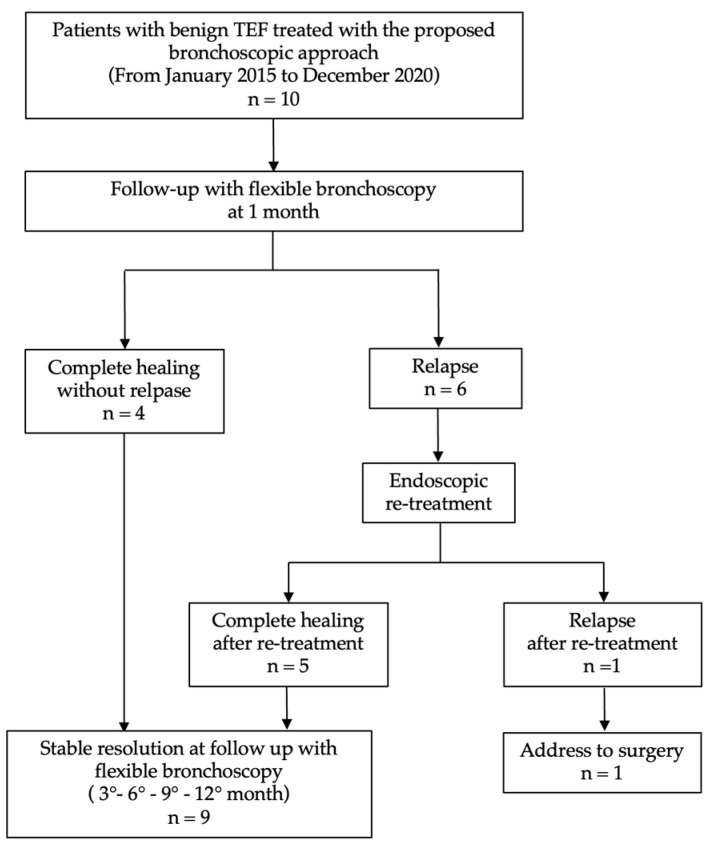
Study flowchart.

The suture was made with either absorbable (Vycryl 3.0, Ethicon, Inc, Raritan, NJ, USA) or non-absorbable (Prolene 3.0, Ethicon US, LLC. 2022, Somerville, NJ, USA) sutures using a long needle holder (Figure 2a–d). In early fistulas, a reabsorbable wire was adopted, which had a reabsorption time of about 3 months, while in late fistulas, which generally require a longer time to heal, a non-absorbable suture wire was preferred. All patients underwent a follow-up examination scheduled for 1 month after the intervention and then at regular intervals of 3 months to 1 year using a flexible bronchoscope (Figure 3).

#### 2.2.3. Post-Procedure Management

After the procedure, patients were followed up for at least 1 h in the recovery room before being sent back to the ward, depending on whether there were complications or not. The continuous monitoring of oxygen saturation was ensured. A chest X-ray and a complete blood count test were obtained after 3 h. Flexible bronchoscopies were performed after 1 month and then scheduled at 3rd, 6th, 9th, and 12th months, to control the suture line.

### 2.3. Outcomes

The coprimary outcomes were the efficacy of the proposed technique in terms of the proportion of fistulas successfully closed at 1 year of follow-up and the rate of procedure-related complications. Adverse effects were classified according to the Clavien–Dindo system [11].

### 2.4. Statistical Methods

Statistical analysis was performed using descriptive statistics and frequency analysis. Demographic, clinical, and procedural data were summarized using the mean, standard deviation, median, or counts and percentages, according to their type. All statistical calculations were performed with R-Software, version 4.4.0 (24 April 2024). The design, conduct, analysis, and reporting in this study conform to the Standard of Reporting Diagnostic Accuracy Guidelines [12].

## 3. Results

From January 2015 to December 2022, 10 patients with non-neoplastic TEFs were treated in our department. The patients were between the ages of 18 and 81 years. Demographic and clinical data are reported in Table 1. In our population, the causes of TEFs were intubation (80%), trauma (10%), and related to esophagectomy (10%) in a patient with a previous esophageal cancer. This patient had previously been treated with an esophageal stent placement, which subsequently dislodged. The most frequent symptom was a cough, which was present in nearly all patients (90%), followed by dyspnea (70%). In these 10 patients, 17 endoscopic repair procedures were performed in total. The mean operative time was 38.6 ± 12.2 min.

### Outcomes: Success Rate and Procedure-Related Complications

Seventeen endoscopic repair procedures were performed in total. Four of the ten patients (40%) presented the complete and durable healing of the fistula without relapse after the first procedure (Table 2). Nine patients (90%) completely healed within 1 year of follow-up. Five patients presented a good clinical result at 1 month, but the partial reopening of the fistula occurred 1 to 3 months after the procedure, requiring a further endoscopic resuture. Of these, one patient experienced a further reopening of the fistula six months after the procedure, which required a further endoscopic resuture. Only one patient presented a complete reopening of the fistula one month after the procedure and was successively referred for surgery. This patient had previously been treated with an esophageal stent, which later dislodged, developing a neoesophagus.

**Figure 4:**
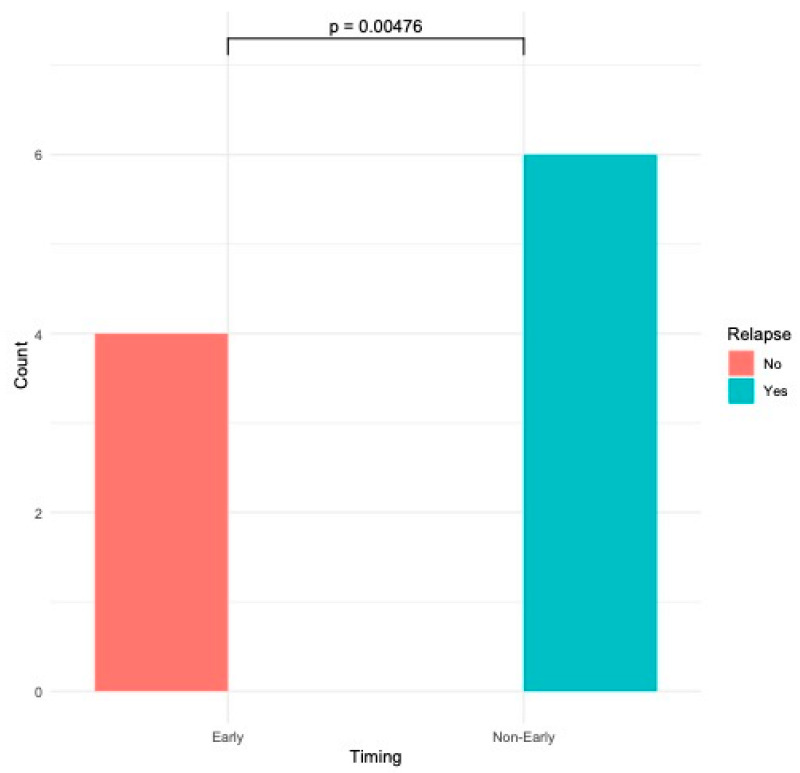
Association between fistula appearance (early vs. non-early) and rate of relapse (Fisher’s exact test).

None of the patients experienced any complications during the procedure or in the post-operative period, and oral nutrition was restored in 1 to 3 days, without adverse effects. Patients with early fistulas, characterized by fragile mucosa with hyperemia, congestion, and fringed margins, had a significantly higher likelihood of long-lasting healing after the first procedure in comparison to fistulas without these macroscopic features at the first bronchoscopy (4 versus 0, respectively; Fisher’s *p*-value < 0.005—Figure 4). The time from symptom onset to endoscopic intervention (onset-to-door) was lower in patients with early fistulas compared to patients with late fistulas [mean: 1.25 (±0.5) months versus 2 (±0.63) months, respectively].

## 4. Discussion

This single-center retrospective study demonstrates the efficacy and safety of an innovative endoscopic technique for the treatment of non-neoplastic TEFs. The proposed method achieved a high rate of fistula closure, with 90% of patients showing full healing within one year. The results suggest that this minimally invasive approach represents a promising alternative to more invasive surgical methods, especially for patients refusing surgery or those considered unsuitable for a surgical approach.

One of the main advantages of this technique is the ability to achieve the lasting closure of fistulas in most cases. This study reports that 40% of patients reached full recovery after the first procedure, highlighting the potential efficiency of this endoscopic approach as a first-line treatment. In patients with fistula reopening, further interventions have been effective in most patients (90%), emphasizing the reproducibility and adaptability of the technique. In only one patient did the complete reopening of the fistula require conversion to surgery. This unsatisfactory result may be explained by the presence of a neoesophagus due to a previously resected esophageal neoplasm.

The higher success rate observed in early fistulas, characterized by hyperemic, congested, and fragile mucosa, is particularly significant. Early fistulas had a significantly higher probability of long-term closure after the first endoscopic suture than late fistulas (4 versus 0, *p* < 0.005). This result suggests that the biological and morphological state of the fistula at the time of the procedure plays a crucial role in outcomes. The time interval between symptom onset and the endoscopic procedure (onset-to-door) has also been evaluated. Patients with an early fistula had a mean onset-to-door time of 1.25 (±0.5) months compared to 2 (±0.63) months in patients with a late TEF. This suggests that patients with a suspected TEF undergoing bronchoscopy in less than about 40 days from symptom onset may have a reasonable higher probability of an early TEF and, consequently, better outcomes. However, the data need to be confirmed in larger-cohort studies.

The endoscopic suture technique has several technical advantages, allowing the precise manipulation and closure of the fistula. The use of a slipknot, such as the Roeder knot, ensures a safe approach to the fistula margins, minimizing the risk of tissue damage. In addition, the flexibility in suture materials—resorbable and non-resorbable—offers an additional level of customization to the patient’s needs and fistula healing requirements. In this respect, we observed that, in patients with a late fistula, non-absorbable sutures appear to be superior in terms of the success rate. This can be explained by there being less active physiological healing mechanisms in patients with late fistulas compared to patients with early fistulas, requiring a longer time for fistula cicatrization. This underlines the importance of early intervention and suggests that this technique could be particularly useful if performed promptly after fistula diagnosis.

Methods for improving and accelerating fistula healing have recently been investigated. Interestingly, the preliminary data obtained using a beagle model suggest that the local administration of mesenchymal stem cells may accelerate the fistula healing process, modulating apoptosis and the inflammation response via the suppression of the TLR4/NF-kB pathway [13]. Likewise, Han and colleagues reported that the local injection of platelet-rich plasma around the fistula using an injection needle succeeded in healing a TEF in a patient with a previously resection for esophageal cancer [14].

Importantly, our technique does not require a tracheostomy, which may be a key consideration for patients considered unsuitable for surgery. The use of Nd–YAG laser for cutting the suture wire facilitates the completion of the procedure and also ensures minimal thermal damage to the surrounding tissues. The procedure, with a mean operating time of 38.6 ± 12.2 min, demonstrates efficiency without compromising accuracy.

Several endoscopic approaches to the treatment of TEFs are reported in the literature, including the insertion of esophageal or combined tracheal and esophageal stents, the application of colloids (fibrin glue or cyanoacrylate), and closure by laser photocoagulation [2,15,16,17,18,19]. Although these treatments are good options for the temporary improvement of symptoms and for the containment of ongoing pulmonary TEF complications, they are predominantly palliative interventions, to provide temporary relief without necessarily solving the problem permanently. Furthermore, the complications of stenting that must be considered include bleeding, stenting displacement, excessive granulation tissue, mucostasis, and poor patient tolerance [20,21].

The method that we propose is, instead, based on a targeted therapeutic strategy, aimed at eliminating the anatomical and functional defect underlying TEFs and minimizing relapses and post-operative complications. In this respect, the safety profile of our method is another significant advantage. No patient reported any complications during or after the procedure. In addition, the recovery of oral nutrition was extremely short, from 1 to 3 days. This contrasts positively with the surgical approach, often associated with increased morbidity and prolonged hospitalization.

We acknowledge that this study has some limitations. The retrospective design of the study and the monocentric nature may limit the generalizability of the results. In addition, the small sample size, although representative of the rarity of non-neoplastic TEFs, means that caution should be exerted in interpreting the data. The lack of a control group treated with alternative techniques represents an additional methodological limitation, which could be addressed via future prospective and multicenter studies. Despite the high success rate, the 50% recurrence rate in the first 3 months, which required further endoscopic interventions, highlights the need to refine the technique to ensure consolidated results. Specifically, non-early fistulae did not show complete healing at the first intervention, highlighting the need for additional strategies for these complex cases. Finally, the one-year follow-up, although adequate to assess short-term results, does not allow any definitive conclusions on the stability of long-term outcomes.

## 5. Conclusions

The results of this study show that the endobronchial suture of TEFs through rigid bronchoscopy without the need for tracheostomy may be a safe and effective first-line option for the management of non-neoplastic TEFs, with a positive impact on healing and safety. This method combines precision, adaptability, and a favorable safety profile, offering a minimally invasive alternative to traditional surgical repair or other palliative endoscopic approaches. Further research and wider implementation are needed to consolidate its role in clinical practice. Future developments could include the integration of advanced suture materials, regenerative technologies, and standardized protocols into complex fistula management. Prospective and multicenter studies with larger samples, controls, and a more extensive follow-up period may provide further evidence of the effectiveness and stability of the results.

## Figures and Tables

**Table 1:** Demographic and clinical features.

Total	*n* = 10
Age mean (SD)	45.1 (±9.8)
Gender	*n* (%)
Male	7 (70%)
Female	3 (30%)
Smoking	*n* (%)
Smokers	6 (60%)
Non-smokers	4 (40%)
Cause of TEF	
Post-intubation	8 (80%)
Trauma	1 (10%)
Post–esophagectomy (esophageal cancer)	1 (10%)
Symptoms	*n* (%)
Cough	9 (90%)
Dyspnea	7 (70%)
Hemoptysis	6 (60%)
Dysphagia	6 (60%)
Sialorrhea	5 (50%)
Comorbidities	*n* (%)
COPD	5 (50%)
Diabetes mellitus	3 (30%)
Systemic arterial hypertension	6 (60%)
Ischemic heart disease	2 (20%)

**Table 2:** Outcomes and complications.

Patients (*n*)	Total (*n* = 10)
Complete healing (without relapse)	4 (40%)
Relapse	6 (60%)
○ Endoscopic re–treatment	5 (83%)
○ Complete reopening	1 (17%)
(referral for surgery)	
Complications	
○ Massive bleeding	n = 0
○ Mortality during the procedure	n = 0

## Data Availability

The original contributions presented in this study are included in the article. Further inquiries can be directed to the corresponding author.
